# Effect of Mitochondrial and Cytosolic FXN Isoform Expression on Mitochondrial Dynamics and Metabolism

**DOI:** 10.3390/ijms21218251

**Published:** 2020-11-04

**Authors:** Mauro Agrò, Javier Díaz-Nido

**Affiliations:** Centro de Biología Molecular Severo Ochoa (CSIC-UAM) and Departamento de Biología Molecular, Universidad Autónoma de Madrid, Nicolás Cabrera, 1, 28049 Madrid, Spain; mau.agro@gmail.com

**Keywords:** Friedreich’s ataxia, mitochondrial frataxin, cytosolic frataxin, mitochondrial dynamics, mitochondrial metabolism, subcellular cross-talk

## Abstract

Friedreich’s ataxia (FRDA) is a neurodegenerative disease caused by recessive mutations in the frataxin gene that lead to a deficiency of the mitochondrial frataxin (FXN) protein. Alternative forms of frataxin have been described, with different cellular localization and tissue distribution, including a cerebellum-specific cytosolic isoform called FXN II. Here, we explored the functional roles of FXN II in comparison to the mitochondrial FXN I isoform, highlighting the existence of potential cross-talk between cellular compartments. To achieve this, we transduced two human cell lines of patient and healthy subjects with lentiviral vectors overexpressing the mitochondrial or the cytosolic FXN isoforms and studied their effect on the mitochondrial network and metabolism. We confirmed the cytosolic localization of FXN isoform II in our in vitro models. Interestingly, both cytosolic and mitochondrial isoforms have an effect on mitochondrial dynamics, affecting different parameters. Accordingly, increases of mitochondrial respiration were detected after transduction with FXN I or FXN II in both cellular models. Together, these results point to the existence of a potential cross-talk mechanism between the cytosol and mitochondria, mediated by FXN isoforms. A more thorough knowledge of the mechanisms of action behind the extra-mitochondrial FXN II isoform could prove useful in unraveling FRDA physiopathology.

## 1. Introduction

Friedreich’s ataxia (FRDA) is an inherited autosomal recessive disease characterized by degeneration and/or a defective development of the spinocerebellar system [1]. The development of the disease is slow but steady, and is characterized by a broad range of clinical symptoms including lack of motor coordination, defective gait, imbalance, dysarthria, diplopia, dysphagia and nystagmus [2]. In addition to these symptoms, a high number of patients also show extra-neurological signs, such as glucose intolerance [3] and hypertrophic cardiomyopathy [4]. In more than 95% of cases, the disease is caused by an aberrant expansion of the GAA (guanine-adenine-adenine) triplet contained in the first intron of the frataxin (*FXN*) gene [5,6] of up to 1700 repeats. The GAA triplet expansion causes a significant reduction in the transcription levels and a gradual heterochromatization of the *FXN* gene [7]. Frataxin is a ubiquitous protein with major abundance in tissues with high energetic demand, such as brain, heart, liver, pancreas and spinal cord. In its canonical mature form, frataxin localizes inside the mitochondrial compartment [8]. It is synthesized as a precursor protein (1–210aa, 23 kDa) that is then sent to the mitochondrion and cleaved twice by the mitochondrial processing peptidase (MPP) [9,10]. This process is mediated by the mitochondrial targeting sequence (MTS) that the precursor possesses on the N-terminal domain. Two proteolytic cleavages produce first the intermediate (42–210aa, 19 kDa) and then the mature form of frataxin (81–210aa, 14 kDa). It is fundamental to understand frataxin functions inside the cell and, even though there still is some debate on its exact roles, the general consensus is that it participates in iron metabolism and iron-sulfur cluster (ISC) formation and regulation. There is strong evidence that frataxin deficiency alters ISC production, reduces the activity of ISC-containing enzymes, induces pathological iron accumulation in mitochondria, and decreases mitochondrial biogenesis, altering mitochondrial metabolism [11,12,13,14,15]. Aside from being the energetic powerhouse of the cell, mitochondria are dynamic organelles able to rearrange themselves, through phenomena called fusion and fission, to properly adapt to cellular metabolic needs. Mitochondrial fusion is responsible for the connection of the mitochondrial network inside the cell, allowing for metabolites, which occur in metabolically active cells that are able to generate ATP more efficiently, to flow rapidly along the network. On the contrary, fission is responsible for fragmenting the mitochondrial network, which is normally associated with resting cells, or disposing of damaged organelles through autophagy processes [16,17]. Furthermore, there is evidence that cells can rearrange their mitochondrial architecture to adapt to changes in metabolic demands [18]. The balance between these states is crucial for maintaining a physiological mitochondrial network. Frataxin deficiency has been widely linked to abnormal mitochondrial structure both in vitro and in vivo [19,20,21,22], showing that frataxin deficiency leads to mitochondrial fragmentation [23,24].

Over time, the existence of a pool of frataxin isoforms has been determined by different groups. The first study suggested that the presence of a cytosolic and nuclear form of frataxin originated through alternative splicing events [8], while another study described an isoform (A1) that originated through alternative splicing, and differed in 11 amino acids at the carboxy-terminal from the canonical frataxin sequence [25]. However, no functional studies were performed on these newly found isoforms until the work of Condò et al. [26]. In that work, an artificial frataxin mutant construct lacking the N-term containing the MTS was generated. This construct was able to localize in the cytosol and conferred protection against pro-apoptotic stimuli induced by ceramide or staurosporine in cells of lymphoid lineage and neuroblastoma. Recently, another group described two more isoforms with different cellular localization and tissue distribution [27]. These isoforms are called FXN II and FXN III, and both lack the MTS. FXN II (14,9 kDa) is transcribed from a different starting point located at a different initial exon (Exon 1b /∆18), while FXN III (18,2 kDa) is obtained through alternative splicing of exon 1a, causing a small deletion of 141 nucleotides (exon 1a, ∆141). It was shown that these isoforms are tissue-specific, with Isoform II localized mainly in the cerebellum and Isoform III most abundant in the heart. Furthermore, their cellular distribution seems to be different too, with FXN II being cytosolic and FXN III being nuclear. Isoform II might be involved in protecting ISCs from oxidative damage, while Isoform III might activate the biogenesis of mitochondrial ISCs [27]. Previous work from our laboratory has demonstrated that, by delivering the whole 135kb *FXN* genomic locus to FRDA patient cells, all of the isoforms are detectable in cells from healthy subjects and patients as well as in the mouse cerebellum [28].

In this work, we decided to focus on the functional characterization of FXN isoform II, which is probably more relevant to FRDA pathophysiology, due to its enrichment in the cerebellum [27]. We selectively expressed the mitochondrial and extra-mitochondrial FXN isoforms in different human cellular models derived from healthy donors and FRDA patients. We then analyzed the effect of overexpressing these two isoforms on the mitochondrial physiology and network structure, in order to gain more insight into a possible cross-talk mechanism inside the different subcellular compartments. These results could give us a broader view on the FRDA molecular basis behind mitochondrial dysfunction induced by FXN deficiency.

## 2. Results

### 2.1. Subcellular Localization of Isoform I and II in Patient OE-MSCs and Fibroblasts

**Figure 1:**
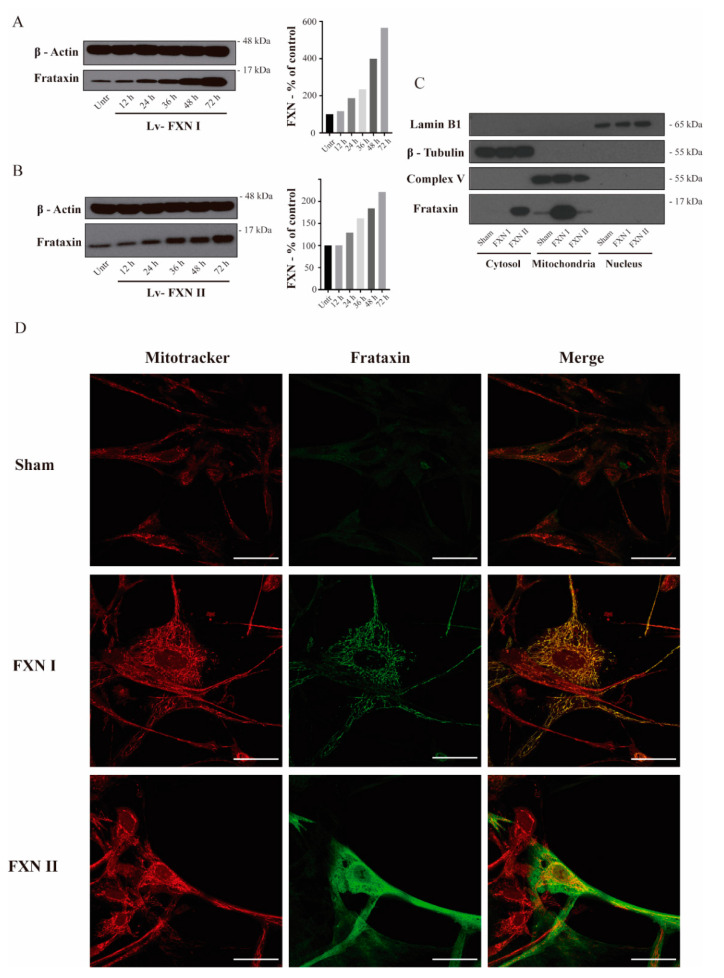
Intracellular localization of frataxin isoform I and II in FRDA-derived OE-MSCs. (**A**) to (**B**) OE-MSCs from FRDA patient transduced for 48 h before cell fractionation with either Lv-FXN I (**A**) or Lv-FXN II (**B**). (**C**) Subcellular fractionation of patient-derived OE-MSCs transduced with lentivectors codifying for FXN isoforms. Specific markers were used to identify the appropriate subcellular compartment: Lamin B1 for nucleus, Complex V (ATP-Synthase) for mitochondrion and β-tubulin for cytoplasm. (**D**) Representative confocal photomicrographs showing FRDA-derived OE-MSCs that were left untreated, or transduced with Lv-FXN I or Lv-FXN II, and stained with Mitotracker (Red) and frataxin (Green). The merged panels show mitochondrial co-localization between frataxin isoform I and Mitotracker, and the cytosolic localization of frataxin isoform II. Calibration bar represents 50 µm.

**Figure 2:**
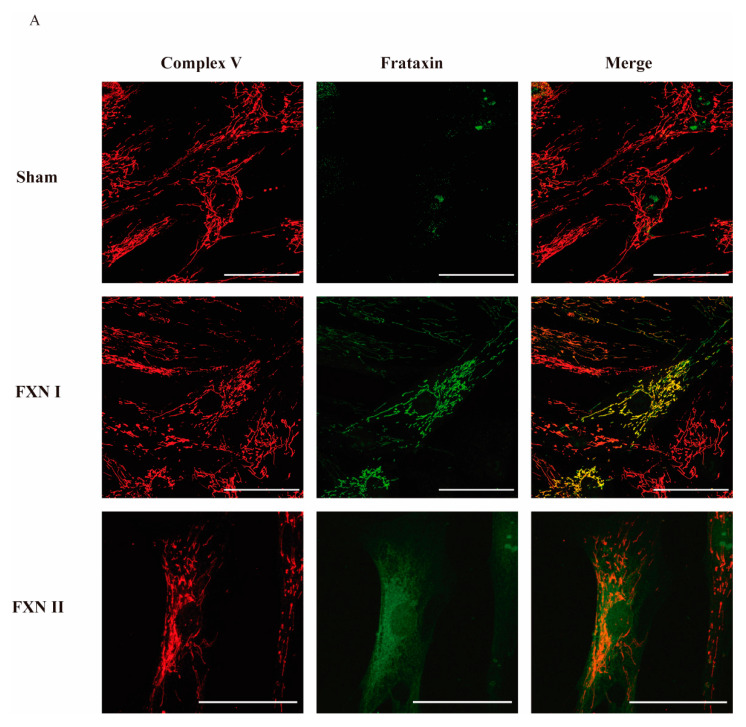
Intracellular localization of frataxin isoform I and II in fibroblasts from FRDA patients. (**A**) Representative confocal photomicrographs showing FRDA-derived fibroblasts either untreated, transduced with Lv-FXN I or Lv-FXN II, and stained with antibodies against Complex V (Red) and frataxin (Green). The merged panels show mitochondrial co-localization between frataxin isoform I and Complex V, and the cytosolic localization of frataxin isoform II. Calibration bar represents 50 µm.

To examine in detail the subcellular localization of frataxin isoforms previously reported in the literature [27], we used two different human cellular models derived from healthy subjects and FRDA patients. Fibroblasts represent a well-established in vitro model for studying FRDA cellular mechanisms [29,30], while olfactory ecto-mesenchymal stem cells (OE-MSCs) are a novel model that has the advantage of being able to be differentiated into several cell types, including neurons [31]. Our lab has extensively worked on the characterization of this cellular model [32]. For this reason, we first transduced OE-MSCs derived from a FRDA patient with the Lv-FXN I (Figure 1A) or the Lv-FXN II (Figure 1B) for 48 h, which allowed us to obtain a frataxin expression level of 200–400% when compared to untreated cells. Then we proceeded with the subcellular fractionation following the protocol described in Materials and Methods [33,34]. To confirm that subcellular compartments were successfully isolated, we examined the expression of Lamin B1 as a nuclear marker, ATP Synthase (Complex V) for mitochondria, and β-Tubulin for the cytosol (Figure 1C). While we did not detect frataxin expression in the nuclear compartment, we were able to confirm the cytosolic localization of FXN II in OE-MSCs, which has a higher molecular weight than the canonical isoform FXN I. To evaluate the cellular localization of the different frataxin isoforms in more detail, we transduced the cells with the two constructs, and analyzed the mitochondrial network and frataxin by immunocytochemistry (Figure 1D). We found that canonical FXN I clearly co-localized with the mitochondrial network of the cells labelled with Mitotracker Red, while FXN II showed a diffuse cytosolic pattern along the cell body. The same conclusions were reached when similar experiments were performed in the other cellular model we analyzed: FRDA-derived fibroblasts (Figure 2A). Cells transduced with Lv-FXN I expressed mature canonical frataxin that co-localized with the mitochondrial network stained with an anti-Complex V antibody, while patient-derived fibroblasts transduced with Lv-FXN II showed a uniform frataxin expression along the cytoplasm of the cell.

### 2.2. Mitochondrial Network Analysis in OE-MSCs and FRDA-Derived Fibroblasts Overexpressing Frataxin Isoforms

**Figure 3:**
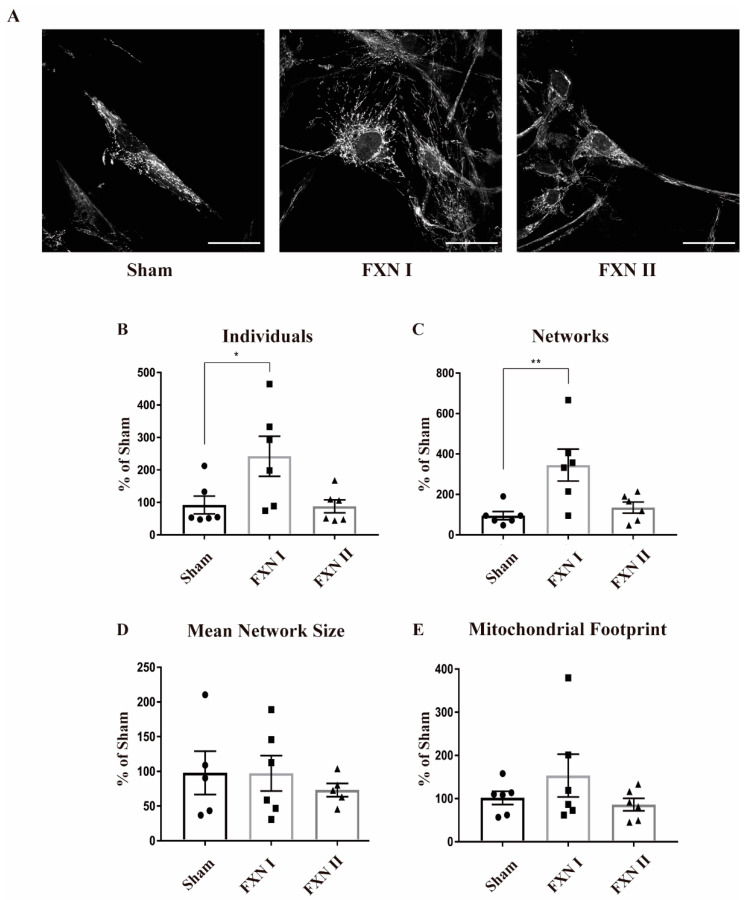
Mitochondrial network analysis in OEMS FRDA patient cells after lentiviral transduction with frataxin isoforms. All cells were stained with Mitotracker and then analyzed by MiNA (Fiji). (**A**) OE-MS patient cells either left non-transduced (left), or transduced with Lv-FXN I (middle) or Lv-FXN II (right) and stained with Mitotracker. Calibration bar represents 50 µm. (**B**) to (**E**) Analysis and quantification of OE-MSc mitochondrial network morphology with MiNA. (**B**) Percentage of raw number of individual mitochondria. (**C**) Percentage of mitochondria arranged in a networked junctioned structure. (**D**) Number of branches per knot. (**E**) Total mitochondrial footprint, indicating the quantity of signal versus background in the image. Data represents mean ± SEM of at least three independent cell culture preparations (*n* = 3). Data were analyzed by one-way ANOVA test with post hoc Dunnett multiple comparisons test. * *p* < 0.05; ** *p* < 0.01.

**Figure 4:**
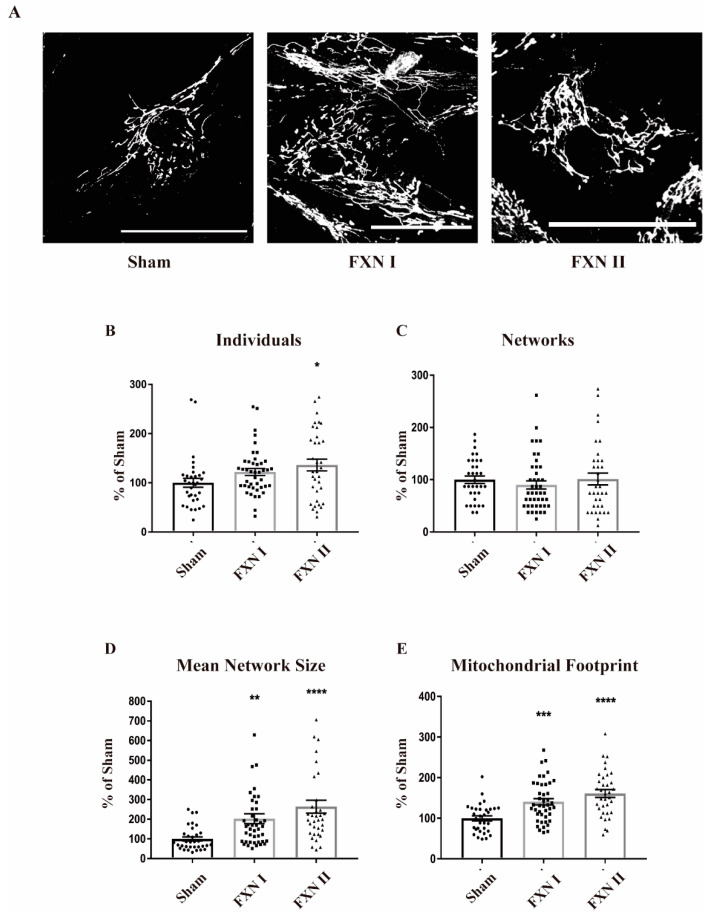
Mitochondrial network analysis in fibroblast FRDA patient cells after lentiviral transduction with frataxin isoforms. All cells were stained with Complex V and then analyzed by MiNA (Fiji). (**A**) Fibroblast patient cells either left non-transduced (left), or transduced with Lv-FXN I (middle) or Lv-FXN II (right) and stained with Mitotracker. Calibration bar represents 50 µm. (**B**) to (**E**) Analysis and quantification of OE-MSc mitochondrial network morphology with MiNA. (**B**) Percentage of raw number of individual mitochondria. (**C**) Percentage of mitochondria arranged in a networked junctioned structure. (**D**) Number of branches per knot. (**E**) Total mitochondrial footprint, indicating the quantity of signal versus background in the image. Data represents mean ± SEM of at least three independent cell culture preparations (*n* = 3). Data were analyzed by one-way ANOVA test with post hoc Dunnett multiple comparisons test. * *p* < 0.05; ** *p* < 0.01; *** *p* < 0.001; **** *p* < 0.0001.

Frataxin plays a crucial role in maintaining mitochondrial metabolic and redox homeostasis. In this regard, mitochondrial dynamics and the fusion/fission balance are tightly intertwined with mitochondrial function [18]. Hence, we wanted to determine if the expression of frataxin isoforms I and II could somehow alter mitochondrial dynamics by modifying their structure, network, branches and/or length of the branches, reflecting some metabolic change induced by frataxin presence. Thus, we analyzed the data regarding mitochondrial network structure with the ImageJ plugin Mitochondrial Network Analysis (MiNA; [35]). To ensure a robust and accurate analysis, we grew the cells in monolayers that prevented overlapping between them, and allowed the analysis of the mitochondrial network in single cells individually. Pictures were taken in Z-stack in order to analyze the whole network. We performed this analysis in FRDA-derived OE-MSCs that were left non-transduced or transduced with Lv-FXN I and II for 48 h, and stained with Mitotracker Red to properly visualize the mitochondrial network (Figure 3A). We observed that transduction with Lv-FXN I increased the number of isolated structures and networked mitochondria (Figure 3B,C), while no changes could be detected in the number of branches per node (Figure 3D) with either frataxin isoform. We also found that transduction with Lv-FXN I induced and increased, albeit not significantly, the mitochondrial footprint (Figure 3E). This result, together with the data obtained about individuals and networks could be associated with a “healthier” mitochondrial network. In contrast, cytosolic isoform II was not able to produce significant changes in mitochondrial structure in this particular cell model (Figure 3B–E). We then analyzed FRDA-derived fibroblasts under similar experimental conditions, that is, we used cells that were left non-transduced as a control or transduced with Lv-FXN I or Lv-FXN II and then stained with an anti-Complex V to visualize the mitochondrial network (Figure 4A). In contrast to what we determined in our OE-MS cell model, we could not detect significant differences in the number of individual or networked mitochondria with the overexpression of frataxin isoform I, even though a small insignificant tendency was observed (Figure 4B,C). However, we found a significant increase in the number of individual mitochondria when transducing cells with cytosolic frataxin isoform II (Figure 4B). Furthermore, significant changes in the number of branches of the network, along with an increment in the footprint were detected with both isoforms (Figure 4D,E). Altogether, these results support the notion that the role of frataxin is highly dependent on the cell type.

**Figure 5:**
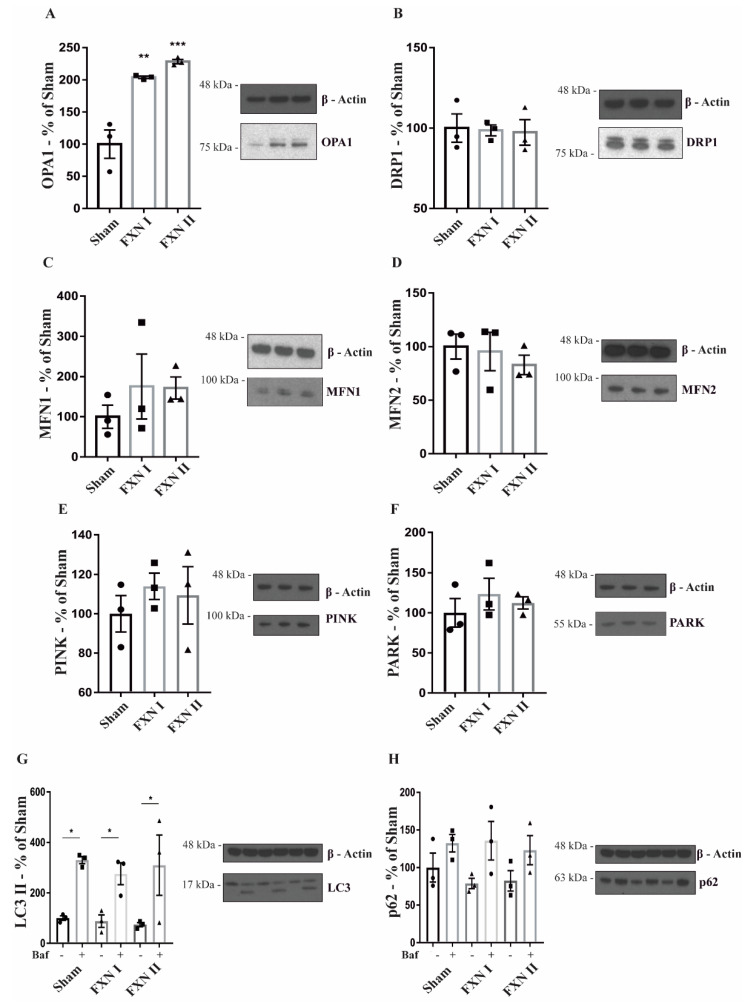
Western blot analysis of mitochondrial dynamics and quality control-related proteins in OE-MS cells. Cells were either left untransduced (Sham) or transduced with Lv-FXN I or Lv-FXN II. (**A**) Analysis of OPA1 levels in OE-MS patient cells. (**B**) Analysis of DRP1 levels in OE-MS patient cells. (**C**) to (**D**) Analysis of MFN1 and MFN2 levels in OE-MS patient cells. (**E**) to (**F**) Analysis of PINK/PARK levels in OE-MS cells. (**G**) to (**H**) Analysis of LC3 and p62 levels with and without autophagy inhibitor bafilomycin A1. Data are represented as mean ± SEM and were analyzed with a one-way ANOVA with post hoc Dunnett multiple comparisons test with *n* = 3. * *p* < 0.05; ** *p* < 0.01; *** *p* < 0.001.

**Figure 6:**
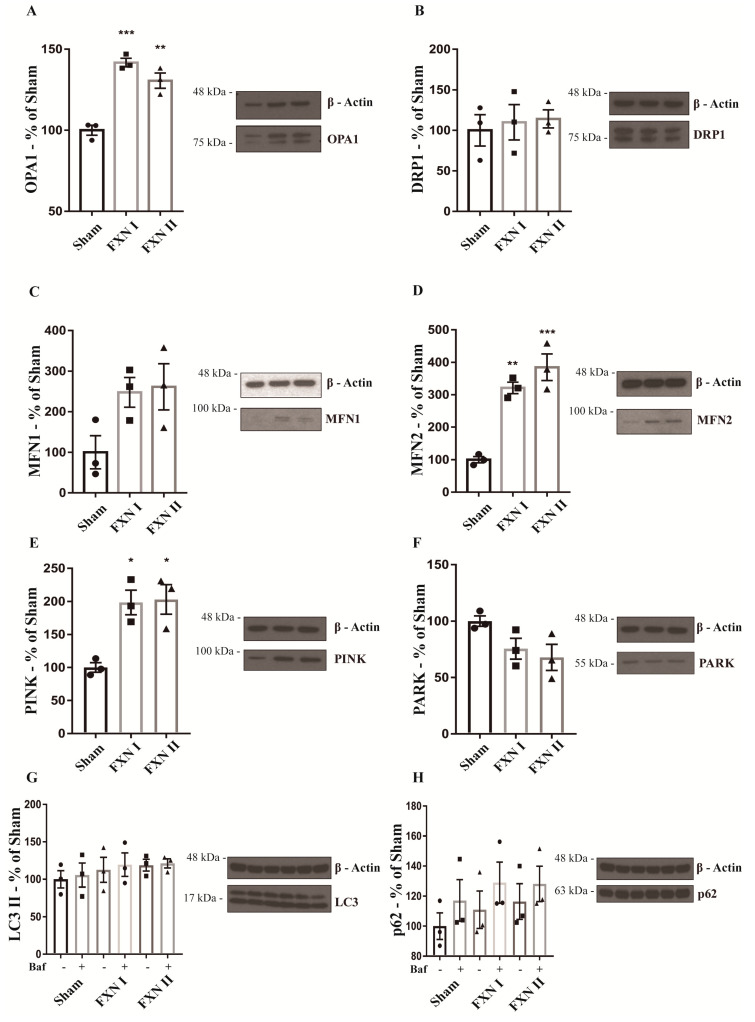
Western blot analysis of mitochondrial dynamics and quality control related proteins in FRDA fibroblast patient cells. Cells were either left untransduced (Sham) or transduced with Lv-FXN I or Lv-FXN II. (**A**) Analysis of OPA1 levels in fibroblast patient cells. (**B**) Analysis of DRP1 levels in fibroblast patient cells. (**C**) to (**D**) Analysis of MFN1 and MFN2 levels in fibroblast patient cells. (**E**) to (**F**) Analysis of PINK/PARK levels in fibroblast patient cells. (**G**) to (**H**) Analysis of LC3 and p62 levels with and without autophagy inhibitor bafilomycin A1. Data are represented as mean ± SEM and were analyzed with a one-way ANOVA with post hoc Dunnett multiple comparisons test with *n* = 3. * *p* < 0.05; ** *p* < 0.01; *** *p* < 0.001.

To confirm the results obtained after the microscopy analysis, we analyzed the expression of several mitochondrial markers through Western Blot, both in OE-MS cells and fibroblasts. In our OE-MS model, we determined a significant increment in the levels of optic atrophy protein 1 (OPA1), one of the principal protagonists in mitochondrial fusion events (Figure 5A), while no significant changes were detected in the levels of the other major players in fusion events, Mitofusin 1 and 2 (Figure 5C,D). Accordingly, no changes were noted in the levels of the major protein involved in mitochondrial fission events, dynamin-related protein 1 (DRP1, Figure 5B). Furthermore, we analyzed the rate of mitophagy by measuring the levels of PTEN-induced putative kinase 1 (PINK-1) and the mitophagic ubiquitine ligase Parkin (PARK-2) responsible for maintaining autophagic control of damaged mitochondria. No increments in mitophagy were detected when transducing OE-MS cells with either FXN I or II (Figure 5E,F). Finally, we analyzed the autophagic flux through the levels of the microtubule-associated proteins 1A/1B light chain 3B (LC3) and the sequestosome 1 protein (p62), both components of the autophagosome, in presence and absence of the autophagy inhibitor bafilomycin A1 (ChemCruz, Cat. No. sc-201550A, 40 nM for 4 h). No changes were detected after transduction with either FXN I or II (Figure 5G,H). In our fibroblast model, we determined a significant increment in the levels of OPA1, MFN2 and PINK with either FXN I or II (Figure 6A,D,E), once again highlighting the impact of cytosolic FXN II on mitochondrial dynamics and quality control.

### 2.3. Mitochondrial Bioenergetics Analysis in Healthy and FRDA OE-MSCs and Fibroblasts

**Figure 7:**
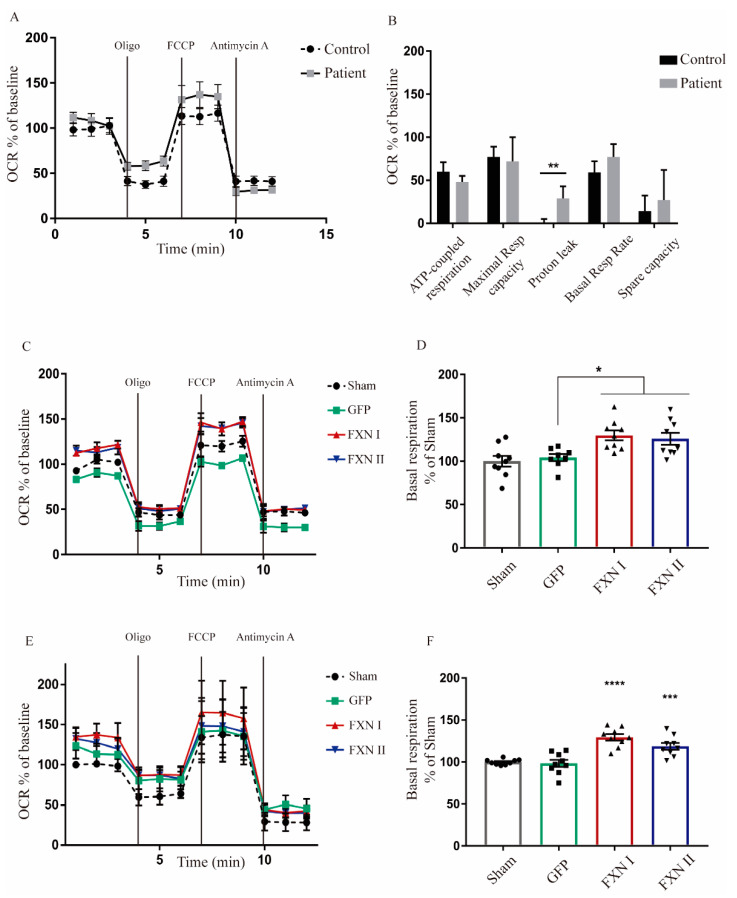
Seahorse assay in OEMS FRDA patients and healthy subjects. (**A**) Seahorse assay performed in untreated healthy and FRDA patient OE-MS cells showing oxygen consumption rates. The X-axis plots different measurements over time (rates). (**B**) Normalization of the seahorse results between healthy and patient OE-MSc showing differences in ATP-coupled respiration rate, maximal respiratory capacity, proton leak, basal respiratory rates and spare capacity. (**C**) Seahorse assay in healthy OE-MS cells either non-transduced or transduced with lentiviral vectors codifying for FXN I, FXN II or GFP as positive transduction control. The X-axis plots different measurements over time (rates). (**D**) Normalization of basal respiration levels in healthy OE-MS cells after transduction with frataxin isoforms. (**E**) Seahorse assay in patient OE-MS cells either non-transduced or transduced with lentiviral vectors codifying for FXN I, FXN II or GFP as positive transduction control. The X-axis plots different measurements over time (rates). (**F**) Normalization of basal respiration levels in FRDA patient OEMS cells after treatment with frataxin isoforms. Data are represented as mean ± SEM and were analyzed with a one-way ANOVA with post hoc Dunnett multiple comparisons test from at least three independent experiments from independent cell culture preparations (*n* = 3). * *p* < 0.05; ** *p* < 0.01; *** *p* < 0.001; **** *p* < 0.0001.

**Figure 8:**
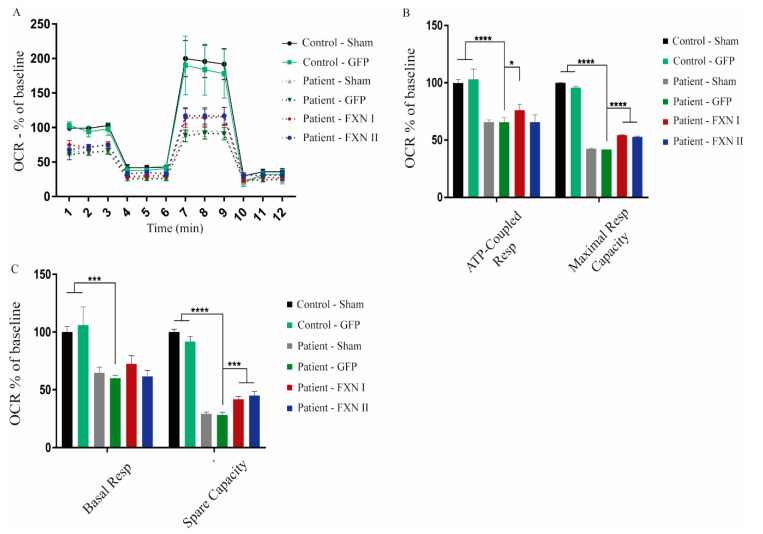
Seahorse assay in fibroblasts isolated from three FRDA patients and two healthy control subjects. (**A**) Representation of the oxygen consumption rate (OCR) in fibroblasts either non-transduced, or transduced with FXN I, FXN II or GFP as a positive transduction control. Patient cells transduced with frataxin isoforms seem to perform better on the assay, getting closer to the healthy cell OCR values. The X-axis plots different measurements over time (rates). (**B**) Relative quantification of the values of ATP-coupled respiration and of maximal respiratory capacity. Significant improvements in maximal respiratory capacity can be detected in patient fibroblasts after transduction with both frataxin isoforms. (**C**) Relative quantification of the values of basal respiratory capacity and spare capacity in both control and patient fibroblasts transduced with both isoforms. Data are represented as mean ± SEM and were analyzed with a one-way ANOVA with post hoc Dunnett multiple comparisons test from at least three independent experiments per cell line (*n* = 3). * *p* < 0.05; *** *p* < 0.001; **** *p* < 0.0001.

After examining the impact of FXN isoforms on the mitochondrial network, we next sought to analyze whether mitochondrial metabolism was affected by the overexpression of FXN isoforms. For this reason, we investigated what was happening at the respiratory chain level through extracellular flux analysis to determine mitochondrial respiration on alive cells (also known as “Seahorse” assays). This technique allows us to measure oxygen consumption rates in basal conditions, adding drugs that target different components of the mitochondrial respiratory chain to assess the fraction of respiration dedicated to ATP production, maximal and spare respiratory capacities and the mitochondrial inner membrane integrity. This approach provides a thorough overview of mitochondrial bioenergetics, giving insight into any kind of dysfunction that may impair mitochondrial metabolism. To characterize and compare their basal mitochondrial metabolism, we started performing this analysis in non-transduced OE-MSCs derived from one healthy subject and one FRDA patient. We found significant differences in the levels of proton leak between patient-derived and healthy control-derived cells. This indicates that, in frataxin deficient cells, protons are able to cross the mitochondrial lipid membrane, migrating to the matrix independently of ATP-synthase, suggesting that their lipid bilayer is damaged (Figure 7A,B). When OE-MSCs were transduced with Lv-FXN I or Lv-FXN II (or Lv-GFP as a transduction control), we detected a significant increase in the basal respiration levels, when compared with GFP-transduced cells, in both healthy (Figure 7C,D) and patient-derived cells (Figure 7E,F). We did not detect any significant change in the levels of ATP-coupled respiration or the maximal respiratory capacity. We then analyzed fibroblasts from FRDA patients and from healthy donors that were transduced with either Lv-GFP, FXN I and FXN II. We compared three different patient cell lines versus two healthy (control) fibroblast lines. Our results showed a clear difference in oxygen consumption rate (OCR) values between the two cell populations. FRDA patient-derived cells visibly showed lower mitochondrial activity, translating into lower OCR values in comparison to healthy cell values, both in their direct measurement and percentage over baseline. When transduced with frataxin isoforms, we observed a modest, but significant, increase in OCR values from patient-derived cells towards healthy OCR values (Figure 8A). The ATP-coupled respiration rate increased when patient cells were overexpressing mitochondrial FXN I when compared with GFP-transduced cells, while no significant effect was detected with cytosolic FXN II. Maximal respiratory capacity seemed to improve when cells were transduced with both isoforms in comparison to GFP transduced ones, thus showing that frataxin is involved in supervising and maintaining mitochondrial homeostasis (Figure 8B). Furthermore, spare respiratory capacity significantly improved in patient cells after frataxin isoforms transduction, while no changes were detected in basal respiration rate (Figure 8C), in contrast to what we determined in the OE-MS model (Figure 7D,F). No changes in proton leak were detected between the two cell lines with or without isoforms suggesting that in frataxin deficiency conditions, the integrity of the mitochondrial membrane is more compromised in OE-MS cells rather than fibroblasts. As mentioned before, these results reinforce the assumption that frataxin specific roles may vary accordingly to the cell model in which they are analyzed.

## 3. Discussion

Recently, different groups have reported the existence of several frataxin isoforms, with functions still not fully characterized, and not strictly localized in the mitochondrial compartment. After the pioneering studies by Campuzano identifying the involvement of frataxin in Friedreich’s Ataxia [5], the idea of the existence of a pool of frataxin isoforms has been gradually explored along the years. One of those works described an alternative transcript containing a different exon 5, codifying for an isoform of frataxin differing from the canonical one for 11 residues at the carboxy-terminal located inside the mitochondrial compartment, since the MTS is located in the first exon [25]. On the contrary, the work of Xia highlighted the presence of frataxin isoforms located in different cellular compartments, differing from the canonical transcript in the first exon, thus lacking the MTS [27]. Other authors have also described an extra-mitochondrial frataxin isoform, firstly characterized in erythrocytes (isoform E), similar to isoform II described by Xia [36], even though the sequences of isoform II and isoform E are different. The existence of a pool of cytosolic frataxin involved in protecting cells from oxidative damage has also been indicated [26]. The presence of cytosolic frataxin isoforms has been recently described in the mouse, being the cytosolic form more abundant than the mitochondrial one in heart, brain and liver. Furthermore, different proteoforms were identified in different tissues, supporting the idea of the existence of different tissue-specific transcripts [37]. We focused our studies on the biological significance of the alternative isoform II described by Xia, mainly due to its enrichment in the cerebellum [27], one of the most affected tissues in FRDA [38,39]. Elucidating the roles of a frataxin isoform that is abundant in one of the most affected brain structures, could be crucial for unraveling the molecular mechanisms of the disease. Moreover, it has been described that the decrease in the level of isoform II in cerebellar samples from FRDA patients with respect to healthy controls is even larger that the decrease in total FXN mRNA [27]. This suggests a potentially important role of isoform deficiency in neurodegeneration in the cerebellum of FRDA patients.

Since frataxin is a key protein for mitochondrial homeostasis, we wanted to study the possible functional differences between FXN I and FXN II in mitochondrial physiology, exploring both network structure and mitochondrial metabolism. There is evidence showing that frataxin overexpression can activate oxidative phosphorylation, which increases the mitochondrial respiration rate in some cellular models [40]. However, it is also known that the overexpression itself can lead to imbalanced biochemical pathways, provoking metabolic dysfunction in several physiological processes in the cell [41,42]. For these reasons, we established an appropriate optimal transduction time of 48 h, allowing frataxin levels to safely rise between 2–4-fold compared to untreated cells, while avoiding potential toxicity derived from sustained overexpression, which has been shown to lead to mitochondrial toxicity in mouse models, starting from 20-fold [43].

One of the roles of frataxin is to participate in the formation and regulation of Fe-S clusters alongside other cellular partners forming the Fe-S assembly machinery. De novo cluster synthesis taking place in mitochondria involves at least 18 known factors including frataxin, scaffold proteins (IscU2), matrix proteins (ISD11) and cysteine desulfurase (NSF1) with the objective of incorporating iron into clusters that are then redirected to their final target [44,45]. Fe-S cluster assembly machinery is not exclusive of the mitochondrial compartment: two different “sets” are present both in mitochondria and in the cytosol. The main components of the Fe-S assembly machinery possess both mitochondrial and cytosolic isoforms [46,47,48]. Interestingly, the mechanism behind the generation of cytosolic/mitochondrial isoforms for such Fe-S components, seems to involve an alternative use of starting codons in the same transcript that translates in a different N-terminal of these proteins, mirroring the same situation for frataxin I and II [27]. Indeed, the cytosolic form of frataxin could be interacting with cytosolic components of the ISC assembly machinery [49]. For these reasons, we have hypothesized that frataxin I and II could reciprocate their functions in their own cellular compartment, establishing a cross-talk that could be crucial for FRDA development. Consequently, we checked for the effect of both isoforms on mitochondrial metabolism and network structure, being the most targeted cellular organelle during iron metabolism impairments caused by frataxin deficiency. After confirming in both cellular models the expected localization of mitochondrial isoform I and cytosolic isoform II, we analyzed their mitochondrial network. In our OE-MSCs model, we detected an increment in the number of isolated and networked mitochondria promoted by mitochondrial isoform I overexpression, while no effects were observed after the overexpression of cytosolic frataxin. Interestingly, the remodeling of the mitochondrial network was accompanied by an increment in the expression of OPA1 levels, one of the major players in mitochondrial fusion events [50], with both FXN I and II, while no changes were detected in the other components of the fusion machinery, MFN1 and 2 [50,51]. Aside from its involvement in mitochondrial fusion, OPA1 is also responsible for the maintenance of the electron transport chain and membrane potential by remodeling of the cristae architecture [52,53,54]. Consequently, Isoform I and II expression improved basal respiration rate: this parameter can increase when substrates are available for the mitochondrion to use or when metabolic pathways are stimulated, indicating an energetic shift in the cell [55]. Taken together, these data suggest that isoform I expression had a direct effect on both the rearrangement of the mitochondrial network and its metabolism, while cytosolic frataxin did not elicit such effects, at least at the evaluated time point. There is evidence that mesenchymal stem cells rely more on glycolytic than aerobic metabolism [56], and that their metabolic preference shifts according to their differentiation state [57,58]. Thus, “dormant” mitochondria in their undifferentiated state that are activated after some stimulus have been shown [59]. In this view, the overexpression of cytosolic frataxin for 48 h could not be enough for mitochondria of FRDA OE-MSCs to transit from their dormant state to the activated one. The increment in OPA1 levels and in basal respiration rate that we detected could be a preliminary step preceding the possible subsequent mitochondrial network rearrangement. Finally, it is worth noticing the difference in proton leak levels caused by FXN deficiency in OE-MS patient cells in comparison to healthy subjects. There is a strong link between the dissipation of proton gradient and the rate of H_2_O_2_ generation [60], with several findings identifying mitochondrial uncoupling as a protective strategy in oxidative stress conditions [61,62,63]. Simultaneously, evidence points to an increase in proton leak and a decrease in membrane thickness induced by reactive oxygen species (ROS) production [64,65]. It is well known that one of the hallmarks of FXN deficiency is an increment in ROS generation [66], thus generating a regulatory feedback between oxygen reactive species and the dissipation of proton gradient in FRDA OE-MS patient cells. In our fibroblast model, oxygen consumption rates between healthy subjects and FRDA patients were clearly different, showing that frataxin deficiency in patients severely impaired mitochondrial metabolism. After the expression of FXN I or II, we observed a shift towards higher levels of OCR in patient-derived cells associated with higher levels of maximal respiratory capacity and spare capacity. Spare capacity is a measure of the ability of the mitochondrion to generate energy in response to cellular stresses and higher rates seem to be associated with higher expression of the complexes of the electron transport chain [67,68]. Indeed, higher levels of spare respiratory capacity have also been linked to stronger apoptosis-resistance mechanisms in a human fibroblast model [69]. Furthermore, mitochondrial isoform I increased the levels of ATP-coupled respiration rate, probably due to its capacity to physically interact and stabilize complex II of the electron transport chain [70]. This translated into changes in the mitochondrial dynamics, since we observed a slight tendency for frataxin isoform I and a significant increase for frataxin isoform II, in the number of individual mitochondria after 48 h. Furthermore, we noted an increment in the number of branches per node, pointing to a thicker network after frataxin overexpression, in comparison to non-transduced cells. These results are supported by the significant increment in levels of the mitochondrial fusion key players OPA1 and MFN2 [50,51], which we highlighted before. Furthermore, we detected a significant increment in the mitochondrial quality control PINK protein with both FXN I and II expression, pointing to a healthier, thicker mitochondrial network. Aside from its most known roles in mitophagy by activation of the PARK pathway [71,72], PINK is also involved in central events of mitochondrial homeostasis. It is responsible for maintaining complex I activity in the ETC [73,74,75] and regulates Ca^2+^ efflux from the mitochondria [76]. It is known that mitochondrial distribution and movement within the cells is mediated by proteins of the cytoskeleton [77]. The remodeling of the network induced by those proteins could be responsible for an increase in mitochondrial metabolism, by facilitating the flux of metabolites along the network [78,79]. Taken together, these data indicate an increase in mitochondrial metabolism in our fibroblast model that is not due to a sheer increment in mitochondrial mass, but to a remodeling of the network itself, probably via OPA1/MFN2, promoting mitochondrial fusion.

## 4. Materials and Methods

### 4.1. FRDA-Derived Fibroblasts

Human fibroblasts isolated from skin biopsies from age-matched and sex-matched healthy subjects (Cat. No. GM08402, GM01652, GM08399) and FRDA patients (Cat. No. GM04078, GM03816, GM03665) were obtained from Coriell Repository (Camden, NJ, USA). Patient age spanned from 13 to 36 years with a mean size of 450 GAA repeats, while control subjects spanned from 11 to 32 years. For experiments, cells were seeded at the appropriate density, as specified later, and cultured in DMEM containing 10% fetal bovine serum (FBS), 0.1 mM non-essential amino acids (NEAA, 44 mM L-Ala, 45 mM L-Asn, 40 mM L-Asp, 40 mM L-Glu, 30 mM L-Pro) and supplemented with 10 ng/mL human recombinant fibroblast growth factor 2 (hrFGF-2, Peprotech, Rocky Hill, NJ, USA, Cat. No. 100-18B) and 10 ng/mL epidermal growth factor (EGF, Peprotech, Cat. No. AF-100-15), adapted from a previously described protocol [80]. Maximum passage number for these cells was 12.

### 4.2. Olfactory Ecto-Mesenchymal Stem Cells

Olfactory ecto-mesenchymal stem cells (OE-MSCs) were obtained from voluntary donors affected with FRDA and from healthy subjects. Biopsies were performed at the “*Gregorio Marañon*” Hospital (Madrid, Spain), following the described protocols [81]. All the donors signed an informed consent and procedures were approved by the ethics committee of the hospital, in compliance with national and EU legislation (No. 6/2006, 27 April 2006). For experiments, OE-MSCs were seeded at the appropriate density in plates previously coated with Matrigel (BD Biosciences, Bedford, USA, Cat. No. 354234) for 30′ at room temperature (RT). Cells were then cultured in CSC medium, composed of DMEM/F12 supplemented with GlutaMAX (Thermo Fisher Scientific, Madrid, Spain, Cat. No. 31331028), 0.5% AlbuMAX I (Thermo Fisher Scientific, Cat. No. 11020013), 10 mM 4-(2-hydroxyethyl)-1-piperazineethanesulfonic acid (HEPES), 0.6% glucose, NEAA, 2% FBS, 1% N2 Supplement (Thermo Fisher Scientific, Cat. No. 17502048), and streptomycin/penicillin mix (100 µg/mL, 100 U/mL). hrFGF-2 (8 ng/mL) and nerve growth factor (50 ng/mL, NGF, Peprotech, Cat. No. 45001) were added freshly each time cells were passaged. Maximum passage number for these cells was 15.

### 4.3. Isoform Plasmids

The plasmid containing the cDNA for human FXN I was a kind gift from Dr. Alexander and Dr. Fleming [82] and is referred to as pLv-FXN I. FXN II cDNA was obtained from PET-II plasmids, kindly donated by Dr. Xia and Dr. Li [27], by restriction digest with enzymes XhoI and XbaI and ligated in the lentivector backbone pLv-FXN I using the same restriction sites. Correct ligation was confirmed by restriction analysis and subsequent sequencing to ensure a correct reading frame.

### 4.4. HIV-1-Derived Lentiviral Vectors

Lentiviral packaging and titration was performed following standard protocols already tested in the lab [83,84]. Three plasmids were used in the lentiviral packaging (2nd generation): a packaging plasmid pCMV dR8.74 (5 µg) containing the viral genes *gag*, *rev*, *pol* and *tat* mandatory for producing viral particles, an envelope plasmid pMD2.G (2 µg) containing the *env* gene needed to pseudotype viral vectors with VSV-G protein, and the plasmid containing the gene of interest (5 µg), including pRRL hCMV/eGFP WPRE from Dr. Naldini’s lab, pLv-FXN I from Dr. Fleming and Dr. Alexander and pLv-FXN II, obtained during this work. These plasmids were transfected into 293T cells cultivated in p100 plates at 80% confluence using the reagent Lipotransfectin (Solmeglas, Madrid, Spain, Cat. No. SBM0959), following the manufacturer′s instructions. After 48 h post transfection, the supernatant was collected and filtered with a low protein-binding 0.45 µm filter, aliquoted and stored at −80 °C until further use. For experiments, cells were transduced with lentiviral vectors for 48 h; this allowed us to obtain around 300% increase in frataxin expression when compared to healthy untreated cells, without affecting cell viability.

### 4.5. Cell Extraction, Subcellular Fractionation and Western Blot Analysis

Cells were plated in M6 multiwells at the optimal density of 200,000/well. After medium removal and washing with ice-cold phosphate buffered saline (PBS), cells were frozen at −80 °C, until used. For subcellular fractionation, the commercial kit Cell Fractionation Kit Standard (Abcam, Cat. No. MS861 [33,34]), was used to separate subcellular compartments, following manufacturer´s instructions. Protein concentration was determined through the BCA assay following manufacturer´s instructions (Thermo Fisher, Cat. No. 23227), reading the absorbance at 562 nm. Then, samples were mixed with loading buffer containing sodium dodecyl sulfate (SDS), boiled for 5′ and proteins were separated through polyacrylamide gel electrophoresis run in 4–12% bis-tris plus gels (Invitrogen, Cat. No. NP0322BOX). Proteins were transferred to a nitrocellulose membrane (iBlot 2 NC Stack, Invitrogen, Cat. No. IB23002) using the iBlot2 transfer device (Invitrogen, Cat. No. IB21001) and then blocked with 5% non-fat dry milk dissolved in PBS and 0.2% Tween 20 (PBS-T). Blocked membranes were incubated overnight at 4 °C with the primary antibody diluted in PBS-T at the concentration recommended by the manufacturer. After primary antibody incubation, membranes were washed 3 times for 5′ each with PBS-T and then incubated with the Horse Radish Peroxidase (HRP)-conjugated secondary antibody of the appropriate host for 1 h at room temperature following manufacturer´s instructions (Southern BioTech, Cat. No. 103005, 403005 and 616005). After this, membranes were washed 3 times for 5′ each with PBS-T and then incubated 1′ with an Enhanced ChemiLuminescence (ECL) solution. The data were analyzed either by a Kodak X-Omat 2000 processor (Kodak, Rochester, NY, USA) or by the biomolecular imager ImageQuant LAS 4000 Mini (GE Healthcare, Chicago, IL, USA). The resulting densitometry data were normalized versus a housekeeping protein: β-Tubulin (1:5000, Sigma, Cat. No. T4026) for cytosol, ATP Synthase Complex V for mitochondria (1:1000, Abcam, Cat. No. ab14730) and Lamin B1 (1:1000, Santa Cruz Biotechnology, Cat. No. sc-30264) for nucleus, in order to reduce the variation derived from the difference between loaded samples. Frataxin signal was detected with a commercial antibody (1:1000, Abcam, Cat No. ab110328). OPA1, DRP1, MFN1, MFN2, PINK, PARK, LC3 and p62 signals were detected with commercial antibodies (OPA1 1:1000, BD Biosciences, Cat. No. 612606; DRP1 1:1000, BD Biosciences, Cat. No. 611113; MFN1 1:1000, Abcam, Cat. No. ab104274; MFN2 1:1000, Abnova, Cat. No. H00009927-M01; PINK 1:1000, Abcam, Cat. No. ab23707; PARK 1:500, Abcam, Cat. No. ab77924; LC3, 1:1000, Sigma Aldrich, Cat. No. LC7543; p62 1:1000, Abnova, Cat. No. H00008878)

### 4.6. Immunofluorescence Staining

Cells for immunofluorescence analysis were cultivated on glass coverslips at the optimal density of 20,000 cells/coverslip. These cells were fixed in 4% paraformaldehyde diluted in PBS for 30′ at RT. For mitochondria staining, before PFA fixation, cells were incubated with 1 µM Mitotracker Red CM-H2Ros (ThermoFisher, Cat. No. M-7513) for 30′. Then, cells were washed 3 times with PBS and blocked for 1 h at RT with a PBS solution containing 1% Bovine Serum Albumin (BSA) and 0.1% Triton-X 100. After blocking, cells were incubated with the primary antibody (FXN, Abcam–ATP Synthase Complex V, Abcam) diluted in blocking solution at the concentration suggested by the datasheet (1:200) overnight at 4 °C. After primary antibody incubation, coverslips were washed 3 times with PBS and incubated with the secondary antibody (1:1000, conjugated with Alexa-488 or Alexa-555, both from ThermoFisher, Cat. No. A-21121 and A-31572) for 1 h at RT, protected from light, washed 3 times with PBS and mounted with the aqueous-based mounting medium Fluoromount-G (Southern Biotech, Cat. No. 0100-01). Images were acquired using either the *LSM510* or the *LSM710* confocal scanning microscope.

### 4.7. MiNA Analysis

Data regarding mitochondrial network structure were analyzed with the ImageJ plugin Mitochondrial Network Analysis (MiNA; [35]). This program allows one to deeply examine the morphology of the mitochondrial network using a quantitative approach, taking advantage of the FIJI platform developed by Schindelin [85]. This analysis allows the estimation of the number of unbranched individual mitochondria and branched networked structures. Furthermore, it allows the detection of the extent of the network formed by mitochondria, how branched the network is, expressed as number of branches per node, and the total mitochondrial footprint, indicating the total area in the image occupied by signal separated from background, thus permitting the estimation of the quantity of fused or fragmented mitochondria. Even though distinct morphologies have been identified for mitochondrial structures, the program allows only for the determination of “individuals” (no-junctioned structures) and “networks” (junctioned structures) and then elaborates the rest of the parameters described before.

### 4.8. Cellular Bioenergetics Analysis

Mitochondrial respiration in real time was analyzed using the SeaHorse XF24 Extracellular Flux Analyzer (Agilent, Santa Clara, USA) with a protocol adapted from Ribeiro et al. [86]. Specifically, we measured the oxygen consumption rate (OCR) per minute in cell cultures subjected to sequential addition of different modulators of respiration, to determine select bioenergetics parameters: the ATP synthase inhibitor 0.5 µM oligomycin (EMD Millipore, Cat. No. 495455), the ionophore and mitochondrial uncoupler 0.75 µM carbonyl cyanide 4-(trifluoromethoxy) phenylhydrazone (FCCP, Sigma, Cat. No. C2920) and the mitochondrial complex III inhibitor 4 µM antimycin A (Sigma, Cat. No. A8674). OE-MSCs and fibroblasts were plated at a density of 2.5 × 10^4^/well in 150 µl of their corresponding seeding medium. Before the experiment, the medium was changed to a special DMEM without sodium bicarbonate supplemented with 1 mM pyruvate and 5 mM glucose. OCR values were used to calculate different parameters of mitochondrial respiration, including basal respiratory capacity, maximal respiratory capacity, spare respiratory capacity, proton leak and ATP-coupled respiration [87]. Three to four points per experimental condition were analyzed in at least three independent experiments per cell type.

### 4.9. Data and Statistical Analysis

No institutional ethical approval was needed for the experiments we performed. Any of the studies treated in this manuscript were pre-registered. No randomization, no blinding and no sample calculations were performed for the experiments described. Due to the exploratory nature of the work, no exclusion criteria were determined. Kurtosis and skewness were analyzed for all data sets to assure Gaussian distribution, allowing for the use of parametric tests. Outliers were searched with Grubbs test. Data were analyzed with Graphpad Prism 7 software with the test indicated in figure legends.

## 5. Conclusions

Several lines of evidence have shown the existence of an extra-mitochondrial pool of frataxin with functions not fully explored yet. In this work, we investigated whether the cytosolic isoform of frataxin, FXN II, could have some impact on mitochondrial physiology through some potential cross-talk mechanism between cytosol and mitochondria. Our data showed, in two different human FRDA cellular models, that the expression of frataxin I and frataxin II altered both mitochondrial respiration, in parameters of basal respiration, maximal respiration and spare capacity rates, the expression of mitochondrial markers and the arrangement of the mitochondrial network. Taken together, these data suggest a potential role for the cytosolic pool of frataxin in the cross-talk between cytosol and mitochondria. Further investigation of the functional role of Isoform II in mitochondrial homeostasis may help us to better understand the contribution of the deficiency of this isoform to neurodegeneration in the cerebellum in Friedreich’s ataxia.

## Abbreviations

ATP	Adenosine Tri-Phosphate
BSA	Bovine Serum Albumine
DMEM	Dulbecco’s Modified Eagle Medium
DRP1	Dynamin-related protein 1
ECL	Enhanced Chemiluminescence
EGF	Epidermal Growth Factor
FBS	Fetal Bovine Serum
FCCP	Carbonyl cyanide-4-trifluoromethoxyphenylhydrazone
FXN	Frataxin
FRDA	Friedreich’s ataxia
hrFGF2	Human recombinant Fibroblast Growth Factor 2
HRP	Horseradish Peroxidase
ISCs	Iron Sulfur Clusters
LC3	Microtubule-associated proteins 1A/1B light chain 3B
MFN1	Mitofusin 1
MFN2	Mitofusin 2
MiNA	Mitochondrial Network Analysis
MTS	Mitochondrial Targeting Sequence
MPP	Mitochondrial Processing Peptidase
NEAA	Not-essential Amino Acids
OCR	Oxygen Consumption Rate
OE-MSC	Olfactory ecto mesenchymal stem cells
OPA1	Optic atrophy protein 1
PARK	Parkin
PBS	Phosphate Buffered Saline
PFA	Paraformaldehyde
PINK	PTEN-induced kinase 1
RT	Room temperature
SDS	Sodium Dodecyl Sulfate
